# High-Performance Anodic Vulcanization-Pretreated Gated P^+^–π–M–N^+^ InAs/GaSb Superlattice Long-Wavelength Infrared Detector

**DOI:** 10.1186/s11671-021-03550-x

**Published:** 2021-05-29

**Authors:** Ju Sun, Nong Li, Qing-Xuan Jia, Xuan Zhang, Dong-Wei Jiang, Guo-Wei Wang, Zhi-Chuan Niu

**Affiliations:** 1 State Key Laboratory for Superlattice and Microstructures, Institute of Semiconductors, Chinese Academy of Sciences, Beijing, 100083 China; 2 Center of Materials Science and Optoelectronics Engineering, University of Chinese Academy of Sciences, Beijing, 100049 China; 3 College of Materials Science and Opto-Electronic Technology, University of Chinese Academy of Sciences, Beijing, 101408 China; 4 Beijing Academy of Quantum Information Sciences, Beijing, 100193 China

**Keywords:** *π* region doping, Gating, Anodic sulphide, Saturation bias voltage

## Abstract

The InAs/GaSb superlattice infrared detector has been developed with tremendous effort. However, the performance of it, especially long-wavelength infrared detectors (LWIR), is still limited by the electrical performance and optical quantum efficiency (QE). Forcing the active region to be *p*-type through proper doping can highly improve QE, and the gating technique can be employed to greatly enhance electrical performance. However, the saturation bias voltage is too high. Reducing the saturation bias voltage has broad prospects for the future application of gate voltage control devices. In this paper, we report that the gated P^+^–*π*–M–N^+^ InAs/GaSb superlattice long-wavelength infrared detectors exhibit different π region doping levels that have a reduced minimum saturation bias at − 10 V with a 200-nm SiO_2_ layer after a simple and effective anodic vulcanization pretreatment. The saturation gate bias voltage is much lower than − 40 V that reported with the same thickness of a 200-nm SiO_2_ passivation layer and similar structure. The optical and electrical characterization indicates that the electrical and optical performance of the device would be weakened by excessive doping concentration. At 77 K, the 50% cutoff wavelength of the device is about 8 µm, the 100% cutoff wavelength is 10 µm, the maximum quantum efficiency is 62.4%, the maximum of responsivity is 2.26 A/W at 5 µm, and the maximum RA of the device is 1259.4 Ω cm^2^. Besides, the specific detectivity of Be 780 °C-doped detector without gate electrode exhibits a peak of 5.6 × 10^10^ cm Hz^1/2^/W at 5 µm with a 70-mv reverse bias voltage, which is more than three times that of Be 820 °C-doped detector. Moreover, the peak specific detectivity could be further increased to 1.3 × 10^11^ cm Hz^1/2^/W at 5 µm with a 10-mv reserve bias voltage that has the bias of − 10 V at the gate electrode.

## Introduction

Type-II strained-layer superlattices (T2SLs) have increasingly become the focus of current research since Sai-Halasz et al. [1] proposed its conception. High-performance infrared detectors can be generated by carefully designing the band structure and strain of T2SLs [2]. InAs/GaSb superlattice, a well-studied member of T2SLs, is an excellent material system exhibiting broad prospects in the infrared detector [3]. The InAs/GaSb superlattice infrared detector has been developed with tremendous effort. However, the performance of it, especially long-wavelength infrared detectors (LWIR), is still limited by the electrical performance and optical quantum efficiency (QE) [4]. The corresponding ambient temperature (ground based) of the LWIR detector is around 300 K, which corresponds to the peak wavelength of 9.6 µm (the center of the LWIR atmospheric transmission window) and has a wide range of applications [5]. It is widely used in various fields such as gas detection, night vision, infrared early warning, infrared remote sensing, and infrared guidance, not only for military use but also for people's life. It is extremely meaningful and challenging to manufacture high-performance long-wave infrared detectors.

The structural design and process preparation of the detector have a significant impact on the performance of the LWIR detector. Increasing the thickness of the active region seemed to be the most direct and effective way to improve the QE. However, more trap centers are introduced along with the increase in the thickness, leading to a reduction in the electrical characteristics of the detector. In the LWIR and very long-wavelength infrared detectors (VLWIR), the InAs layer tends to be thicker than the GaSb layer. Thus, the material is *n*-type (the minority carriers are holes). Forcing the active region to be *p*-type through proper doping can highly increase the QE without any change in the region size of the device [6]. However, it is not the higher the doping concentration, the more the improvement in the device performance. Particularly, the electrical [7] and the optical performance of the device could be weakened by excessive doping concentration.

In addition to changing the doping concentration in the *π* region, the gating technique has been applied in the middle-wavelength infrared detectors (MWIR) and LWIR detectors [8] recently to improve the device performance. However, it requires a very high gate bias voltage. The gate bias can be expressed by Eq. (1).

1 $$\sigma = \varepsilon \varepsilon_{0} V/d,$$

where $\varepsilon$ denotes the relative dielectric constant of the dielectric layer, $\varepsilon_{0}$ represents the dielectric constant of vacuum, *V* refers to the saturation gate bias voltage, *d* is the thickness of the dielectric layer, and *σ* stands for the charge density on the interface. The gate bias has been weakened based on the formula with great effort; the means of using high-k dielectric such as Y_2_O_3_ [9] to passivate or reducing the layer’s thickness [10] is effective. However, there is little research reducing the charge density. In this paper, anodization is first performed to significantly reduce saturation bias. A mixture of NaS_2_·5H_2_O and ethylene glycol is used as the vulcanizing solution. During the anodic curing process, the combination of sulfur atoms and dangling bonds on the surface of the device closes the conductive channels on the surface of the device [11], reduces the surface recombination of the device, and weakens the surface charge density of the device. Then, a layer of dense and uniform elemental sulfur on the surface of the device is obtained. Next, the surface of the elemental sulfur layer is covered with a layer of 200-nm SiO_2_. The gate electrode is placed on the SiO_2_ layer. The saturation gate bias voltage is reduced as the charge density on the interface decreases.

In this study, anodic vulcanization-pretreated LWIR P^+^–*π*–M–N^+^ detectors are manufactured under a low-saturation gate bias voltage based on InAs/GaSb T2SLs with different doping *π* regions. The results indicate that it is not the higher the doping concentration, the more the improvement in the device performance. Specifically, the electrical and optical performance of the device would be weakened by excessive doping concentration. Besides, anodic vulcanization pretreating can significantly reduce the gate bias at − 10 V that is much lower than that reported at the same thickness of a 200-nm SiO_2_ passivation layer with about 40 V in a similar structure.

## Methods

### Materials and Structure

**Fig. 1:**
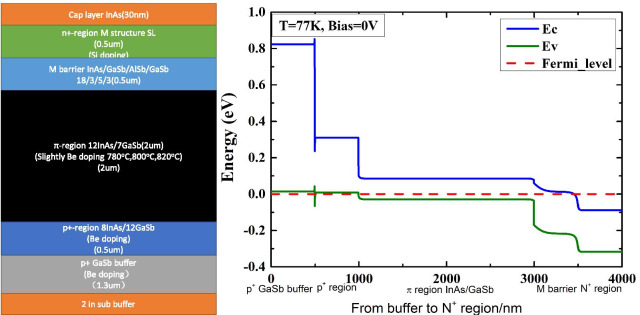
The epitaxial structure and band alignment of the materials with different π region doping levels

**Fig. 2:**
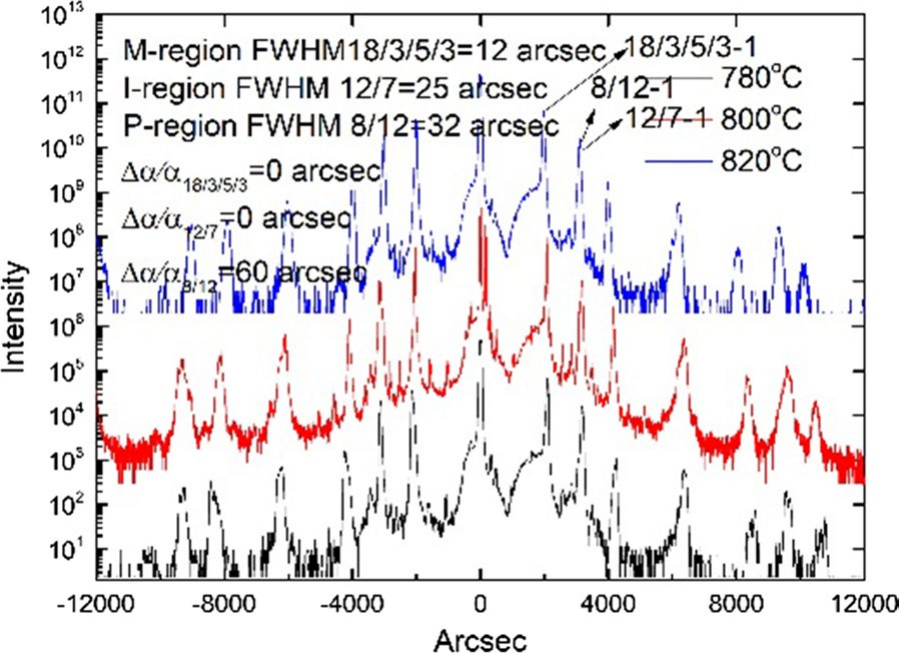
HRXRD rocking curves for samples with different π region doping levels

The materials are produced by a solid source GEN 20 MBE system on the 2-inch *n*-type GaSb (001) substrates. In this work, the high-performance LWIR detector is based on the P^+^–*π*–M–N^+^ structure. A schematic of the devices, high-resolution X-ray diffraction (HRXRD) patterns, and atomic force microscopy (AFM) is illustrated in Figs. 1 and 2. Figure 1 indicates that the structure consists of a 1300-nm-thick P^+^ GaSb buffer, followed by a 500-nm-thick 8-ML InAs/12-ML GaSb (Be: about 1 × 10^18^ cm^−3^)P^+^ region, a 2000-nm slightly P-doped 12-ML InAs (Be: 780 °C 800 °C 820 °C)/7-ML GaSb *π* region, a 500-nm undoped 18-ML InAs/3-ML GaSb/5-ML AlSb/3-ML GaSb M-region, a 500-nm-thick 18-ML InAs/3-ML GaSb/5-ML AlSb/3-ML GaSb (Si: about 1 × 10^18^ cm^−3^) N^+^ region, and a 30-nm N^+^ InAs Cap layer. And it also shows the simulation band alignment with the structure. Considering that the performance of the P^+^–*π*–M–N^+^ LWIR detector would be significantly influenced by the doping of the *π* region, we prepare three samples with different Be doping temperatures varying from 780 to 820 °C.

The superlattice periods of 59.3 Å, 58.4 Å, and 89.5 Å for the *p*-contact, *p*-active region, and M-structure layer, with lattice mismatches of 60 arc sec, 0 arc sec, and 0 arc sec, correspondingly, are exhibited in Fig. 2. The full width at half maximum for the SLs in each region is 32 arc sec, 25 arc sec, and 12 arc sec, indicating that the material has high quality at the interfaces.

**Fig. 3:**
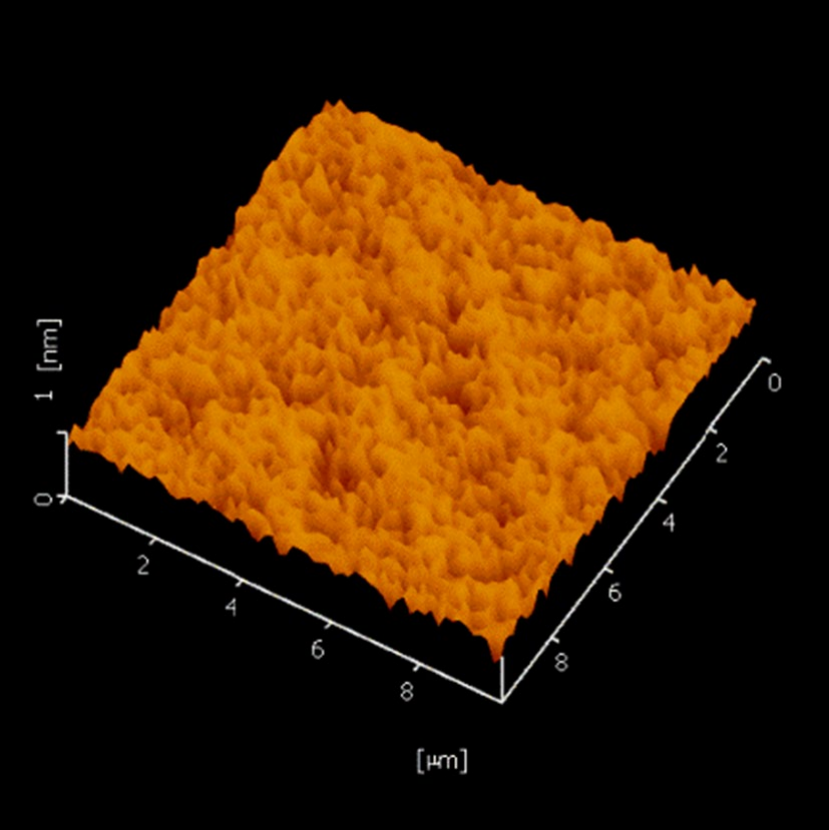
AFM of a 10 × 10 µm^2^ area of a sample

Figure 3 presents that atomic steps appear with the root mean square (RMS) of roughness with 1.87 Å over a 10 × 10 µm area.

### Device Structure and Fabrication

The process is similar to that in Reference [12]. First, the wafer is covered with SiO_2_ as a hark mask. Then, corresponding standard lithography steps are adopted. Then, we do hard mask open by inductively coupled plasma (ICP) system. Next, the mesa is obtained using an inductively coupled plasma (ICP) system with a CH_4_/Cl_2_/Ar mixture. Specifically, the wafers are etched from the top layer to the P^+^ contact [12]. Afterward, the remaining SiO_2_ layer is removed. Then, we immerse one side of the wafer in a mixed solution of sodium sulfide and ethylene glycol and then apply a constant current to the wafer and set a threshold voltage. A sulfur atom layer will be formed on the surface of the film, and the resistance will change. The voltage on the wafer will gradually increase until it reaches the threshold voltage, and then, the vulcanization is completed. Then, the elemental sulfur layer is covered with a layer of 200-nm SiO_2_. Besides, photolithography is performed again to open the window through the layer of SiO_2_ and elemental sulfur as the metal contact regions of the top and bottom metal electrodes. In addition, another photolithography designed with two electrode shapes is conducted; one electrode shape is for the gated diode (GD) and the other is for the ungated diode (UGD). Ti (50 nm)/Pt (50 nm)/Au(300 nm) is deposited by electron beam deposited for the metal electrodes. Finally, the top, bottom, and gate electrodes are acquired by metal lift-off.

**Fig. 4:**
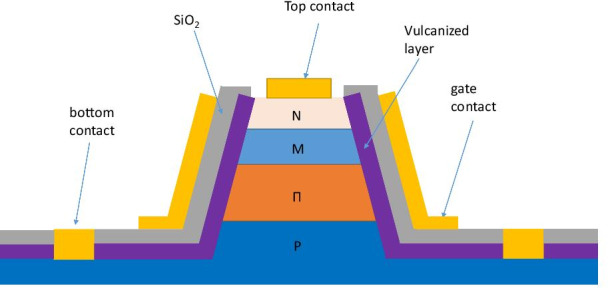
The device structure diagram of GD

Figure 4 illustrates the structure of the GD. As we know, the slope angle of material etching can be adjusted by changing ICP power, RF power, gas flow, and chamber pressure. In this study, the actual inclination angle of the side wall is between 80 degrees and 85° to make it easier for deposit gate contact on the sidewall. The gate electrode is placed on the sidewall of the SiO_2_ layer.

**Fig. 5:**
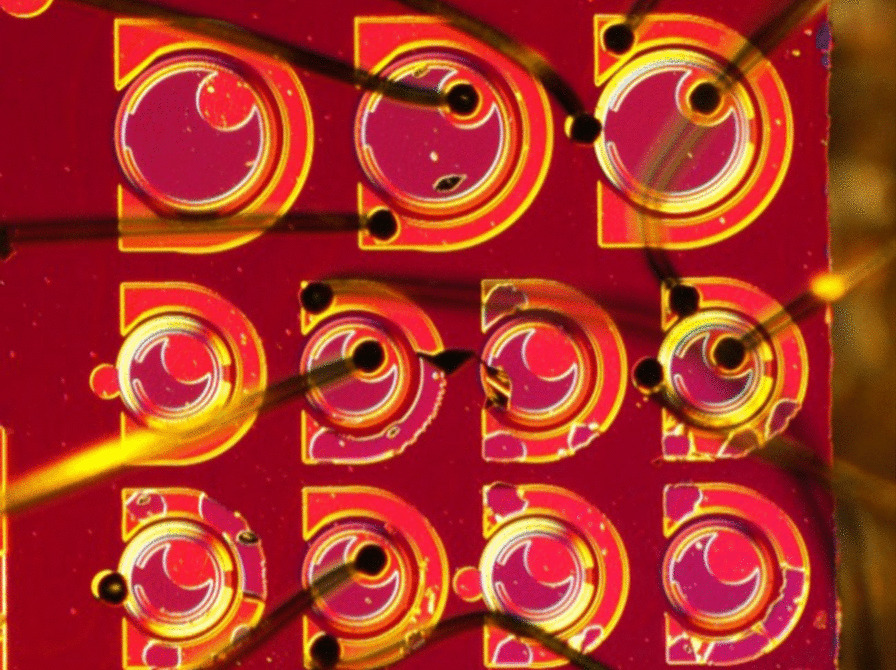
Picture of the device under an optical microscope

Figure 5 demonstrates that half diodes are deposited to be GDs in the three dies (780 °C, 800 °C, and 820 °C Be doping). Then, both the gated diode (GD) and ungated diode (UGD) can be obtained. Ultimately, sample A (780 °C GD), sample B (780 °C UGD), sample C (800 °C GD), sample D (800 °C UGD), sample E (820 °C GD), and sample F (820 °C UGD) can be acquired.

## Results and Discussion

**Fig. 6:**
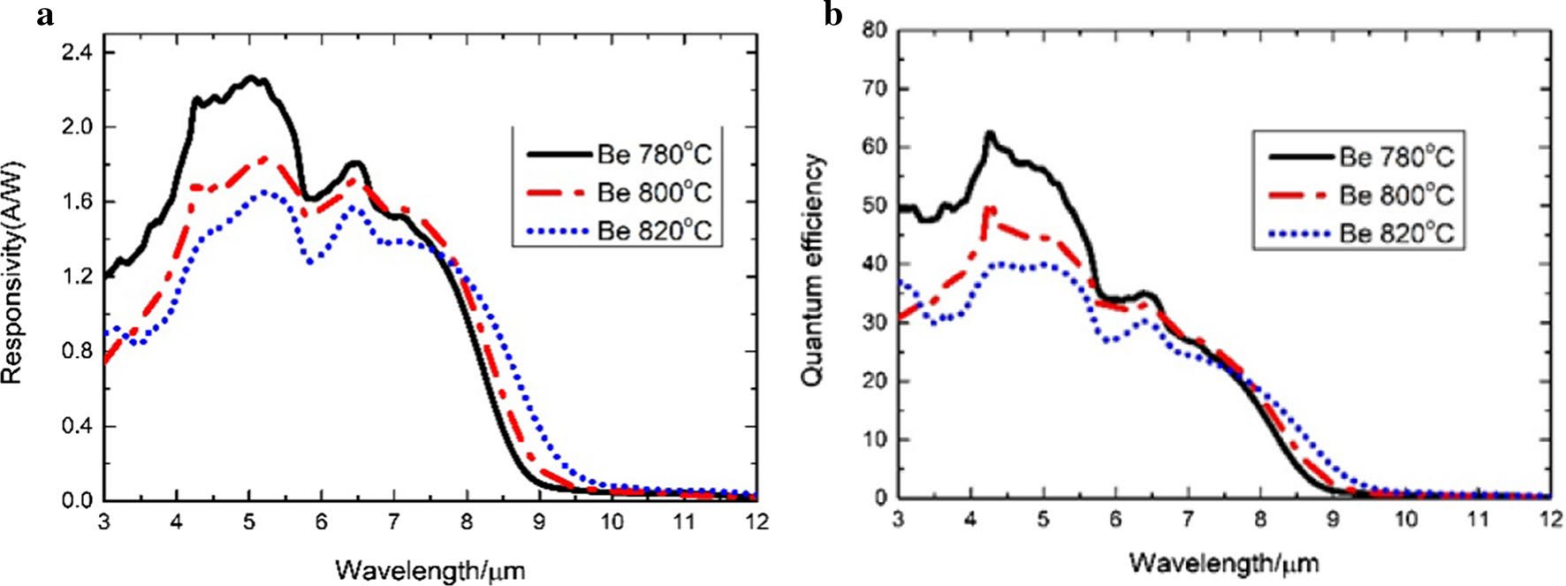
The optical characteristics of the samples with different π region doping levels at 77 K

In infrared detectors, a specific detectivity (D*) is usually used to characterize detector performance, which is calculated by Eq. (2).

2 $$D^{*} = \frac{Ri}{{\sqrt {2qJ + 4\frac{kT}{{RA}}} }}$$

where *q* denotes the amount of electronic charge; *K* refers to the Boltzmann constant; *T* is the Kelvin temperature; Ri refers to the responsivity of infrared detector; *J* is the dark current density of the device under a certain bias; and *RA* refers to the product of resistance value and die area. *J* and RA characterize the electrical performance of the device. And Ri can be converted into QE with the formula:

3 $$QE = \frac{hC}{{q\lambda }}Ri$$

where $h$ is the Planck constant, ${ }C$ is the speed of light, *q* denotes the amount of electronic charge, ${ }\lambda$ is the specific wavelength, and QE and Ri characterize the optical performance of the device. Figure 6 exhibits the optical characteristics of the samples with different *π* region doping levels at 77 K. All samples have similar a 50% cutoff wavelength of 8 µm and a 100% cutoff wavelength of 10 µm at 77 K. Although the QE and responsivity of the device can be increased by changing the doping type to be *p*-type of the *π* region, it is not the higher the temperature, the higher the QE and responsivity. However, the QE and responsivity are significantly reduced with the increase in the doping concentration. For type-II strained-layer superlattices (T2SLs), the doping temperature during growth is critical to the doping concentration. The higher the temperature, the higher the doping concentration. The QE of 780 °C reaches its maximum value of 62.4%, which is 1.5 times larger than that of the QE of 820 °C. It is because too many impurities are introduced with the increase in the doping concentration, leading to the decrease in excess carrier lifetime/diffusion length and the reduction in QE and responsivity [6]. They, furthermore, cause the spectroscopic redshift in Fig. 6a. Figure 6a and b indicates that 780 °C is the best doping temperature of the materials for optical characterization, with the peak responsivity of 2.26 A/W at 5 µm and the peak QE of 62.4%.

**Fig. 7:**
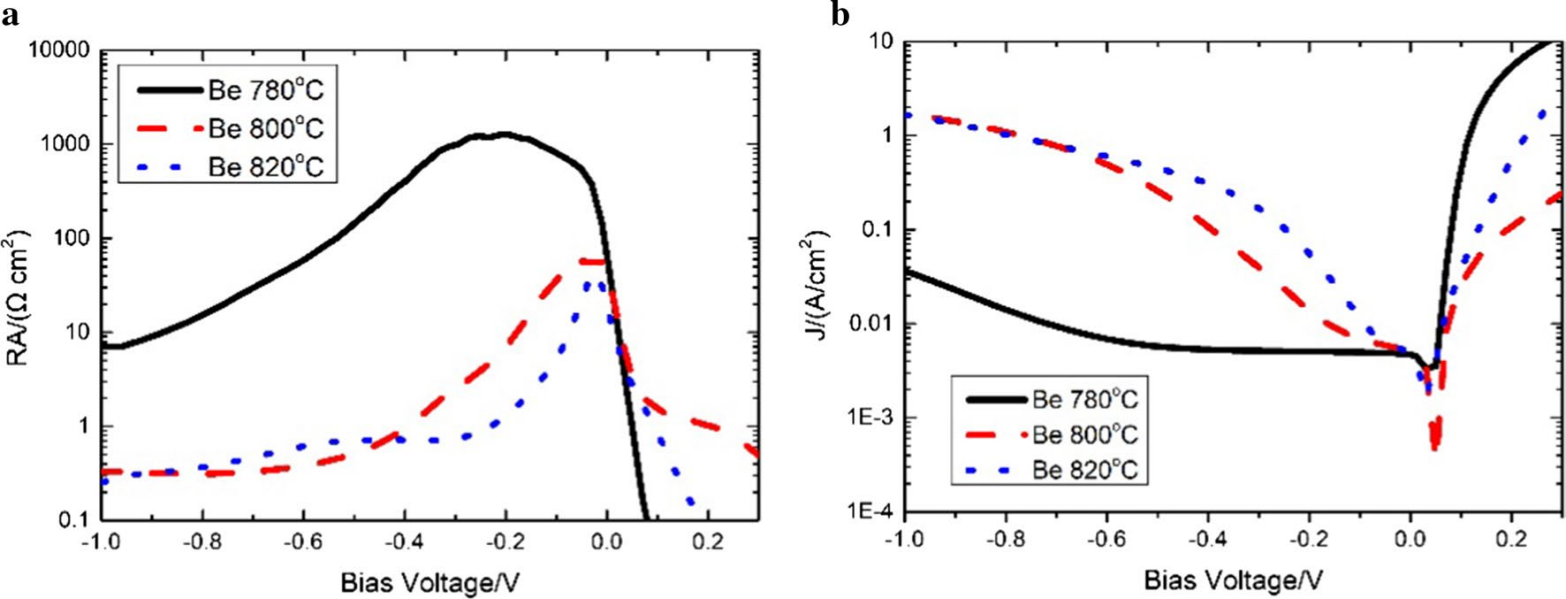
The electrical characteristics of the UGD samples with different *π* region doping levels at 77 K

Figure 7 displays the electrical characteristics of the UGD samples with different *π* region doping levels at 77 K. The electrical performance of the device would be highly affected by different *π* region doping concentrations [7]. With the increase in the π region doping level, the product of resistance value and die area (RA) in certain bias becomes smaller, and the dark current density becomes lager correspondingly. Similar to Reference [6], RA reaches its maximum at nearly 0 V with a soft breakdown as the reverse bias increases, suggesting that the device has a tunneling dark current mode. We reach a RA’s maximum of 1259.4 Ω cm^2^ with Be doped with 780 °C at − 200 mv that is nearly 40 times that of Be doped with 820 °C. Figure 7b illustrates that the dark current density is similar in the negative bias at the range of − 0.1 to 0 V, and the dark current density with Be doped with 780 °C is a little smaller compared to others. The dark current is 4.9 × 10^−3^ A/cm^2^ for the device with Be doped with 780 °C at − 70 mv.

**Fig. 8:**
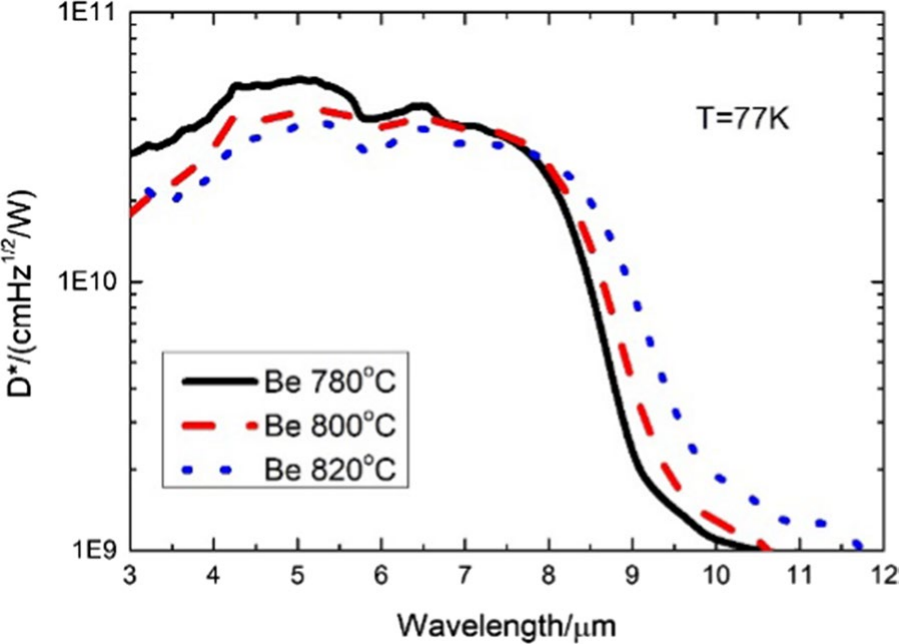
Correlation between the specific detectivity at 77 K of UGD samples with *π* region doping levels and wavelength

The corresponding D* can be calculated by taking the specific Ri, *J*, and RA values under different bias voltages at 77 K. Figure 8 exhibits the detectivity of the UGD samples with different *π* regions. At − 30 mv, the peak detectivity is 5.6 × 10^10^ cm Hz^1/2^/W at 5 µm with Be doped with 780 °C, while it is 3.8 × 10^10^ cm Hz^1/2^/W with Be doped with 820 °C. The peak detectivity of Be doped with 780 °C is 1.5 times that of the Be doped with 820 °C. Therefore, the appropriate doping concentration is extremely significant. However, too high doping concentration would weaken the device performance.

**Fig. 9:**
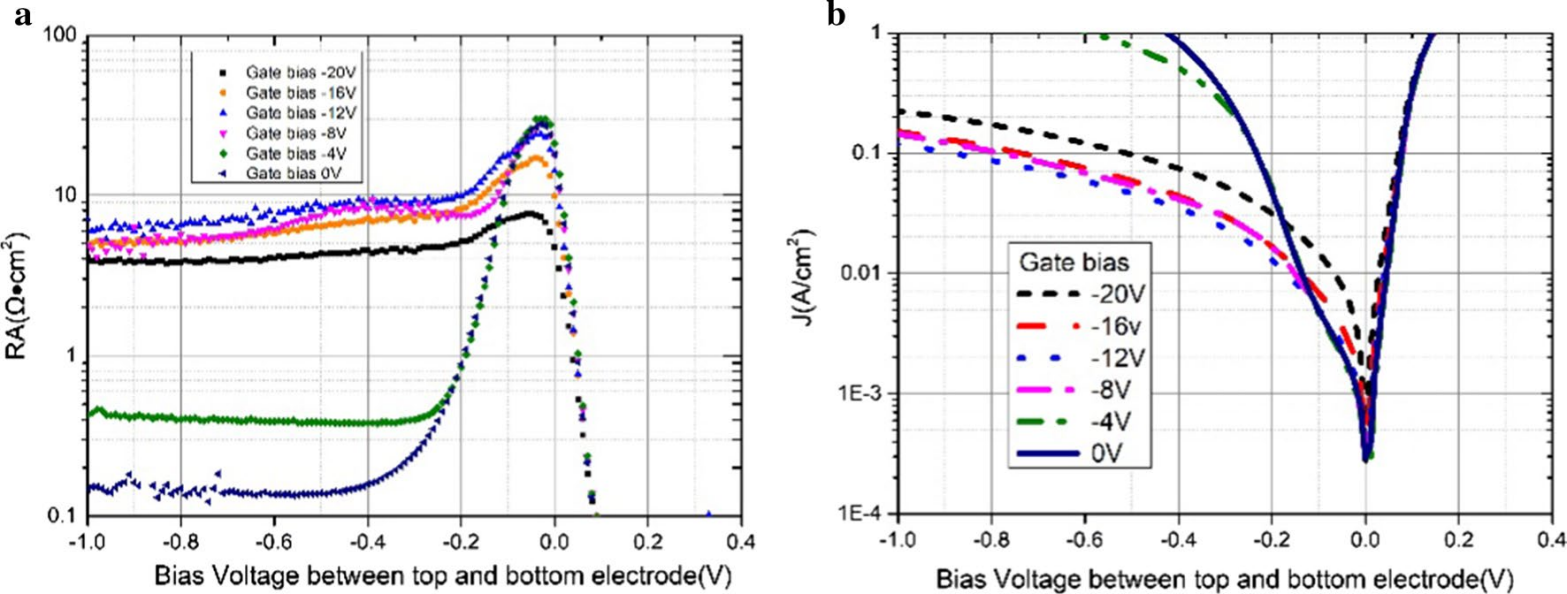
The electrical characteristics of GD samples with 780 °C Be doped with different gate bias voltages and the bias voltage on the top and bottom electrode

**Fig. 10:**
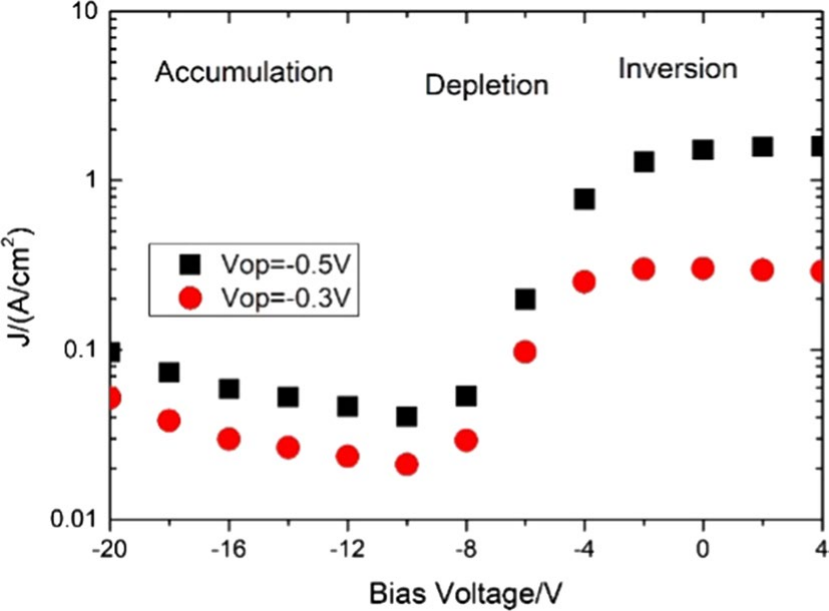
Correlation between the reverse dark current density and the gate bias of GD sample with 780 °C Be doped at different diode operation biases

Figure 9 presents the electrical characteristics of the GD sample with Be doped with 760 °C at 77 K. Different from ordinary gate voltage control devices, anodic vulcanization pretreatment is first performed in this study to remarkably reduce the saturation gate bias voltage. Besides, a mixture of NaS_2_·5H_2_O and ethylene glycol is used as the vulcanizing solution. Anode vulcanization passivation method is employed to form a layer of dense and uniform elemental sulfur on the surface of the device. During the electrochemical reaction, sulfur atoms are combined with the dangling bonds on the device surface, contributing to closing the electronic channels generated by the surface dangling bonds and isolating the device surface electron–hole recombination mechanism [11]. Then, the surface of the elemental sulfur layer is covered with a protective layer of 200-nm SiO_2_, and the gate electrode is placed on the sidewall of the SiO_2_ layer. As reported in previous research, the correlation between the saturation bias and the thickness of the device dielectric layer is nearly linear. Figure 9 suggests that the saturation bias of the device can be reduced at about − 10 V through a simple and effective vulcanization pretreatment; this reduced value in other researches is about 40 V and is four times bigger in devices of similar structure with the same thickness SiO_2_ layer [10]. Besides, RA reaches its maximum of 25 Ω cm^2^ at near 0 V. The downward trend has significantly slowed when we apply negative bias voltage at about − 10 V. When we apply the bias voltage of − 10 V on the gate electrode, RA is 10 Ω cm^2^ at − 0.3 V, which is 40 times that under no applied bias voltage. Besides, it is nearly two orders of magnitude lower than that under no bias voltage at − 0.6 V. Figure 9b indicates that the dark current reaches its minimum of 2 × 10^–4^ A/cm^2^ near 0 V, and it is reduced by an order of magnitude at − 0.3 V. As we know, the IV curve would not change with the gate bias at 0 V when the bias voltage is positive. Besides, the RA of the device increases significantly when the bias voltage increases from 0 to − 10 V; meanwhile, the dark current of the device decreases correspondingly. When the bias voltage varies from − 10 to − 20 V, the RA of the device decreases slightly, and the dark current of the device increases correspondingly. At high reserve bias (e.g., − 1 V) between top and bottom electrodes, dark current decreases with gate bias and then increases slightly beyond − 12 V. It is mainly for gate bias characteristic. Further introduction is shown in Fig. 10. For low reverse bias (e.g., − 0.1 V), the dark current seems increase as gate bias increases, which is completely different from that at − 1 V. For different reverse bias, we suspect that the main leakage mechanism is different. For low reserve bias, high gate bias shows negative influence for it influences surface scattering of electrons and hot electrons. And for high reverse bias, leakage decreases for its surface leakage current decreases. So it is different.

As illustrated in Fig. 10, at high reserve bias (e.g., − 1 V) between top and bottom electrode dark current decreases with gate bias and then increases slightly beyond − 12 V; we can see the device exists in three stages with the change in bias voltage at 77 K [10]. According to Reference [13], the P^+^ and N^+^ regions for P^+^–π–M–N^+^ structure device are heavily doped, and the M region is a lager band region with a larger effective mass compared to the *π* and P^+^ region; therefore, the gate bias has much more influence on the *π* region compared to others [13]. Using a similar method with Chen [10], three stages during the process of high negative bias voltage (− 20 to − 10 V) being applied on the gate electrode are analyzed; the results indicate that the mesa sidewall is on the flat condition or under accumulation [8], and the dark current density slightly increases with the gate bias due to the vulcanization interface. Suspect for vulcanization interface slightly density inhomogeneity, somewhere density insufficient slightly break down. When negative bias voltage (− 10 to − 2 V) is applied, the mesa sidewall gets into depletion, and the dark current increases smoothly. Besides, the field-induced depletion width reaches its maximum and inversion layer formation when the bias voltage at − 2 V is applied to positive gate bias; thus, the dark current density keeps constant. According to Reference [14], why the surface generation–recombination (G–R) current at − 0.5 V is larger than that at − 0.3 V is explained.

**Fig. 11:**
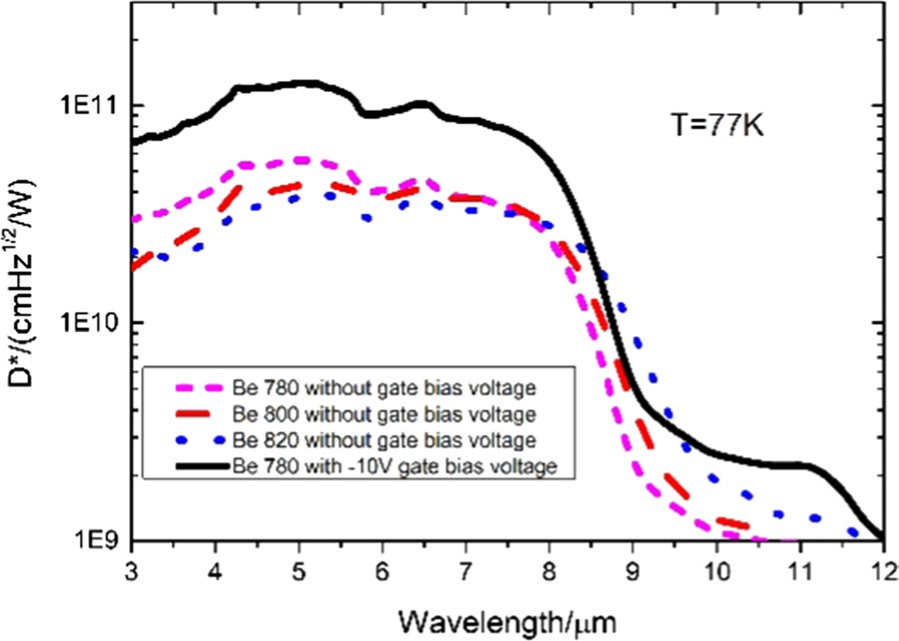
Correlation between the detectivity of the GD sample with 780 °C Be doped and UGD samples with different π region doping levels and the wavelength at 77 K

Figure 11 exhibits that, with the gate bias at − 10 V, the D* for GD sample with 780 °C Be doped reaches its peak detectivity of 1.3 × 10^11^ cm Hz^1/2^/W at 5 µm, which is more than two times that under no bias voltage with 780 °C Be doped and more than three times that under no bias voltage with 820 °C Be doped at 77 K. It indicates that applying appropriate negative bias can significantly improve the device performance.

## Conclusions

2017 Northwestern University reported long-wavelength infrared (LWIR) nBn photodetectors based on InAs/InAs_1−*x*_Sb_*x*_ type-II superlattices. The device exhibited a cutoff wavelength of ∼ 10 µm at 77 K with a peak responsivity of 2.65 A/W, corresponding to a quantum efficiency of 43% and RA 664 Ω cm^2^ and a dark current density of 8 × 10^5^ A/cm^2^, under 80 mV bias voltage at 77 K; the photodetector exhibited a specific detectivity of 4.72 × 10^11^ cm Hz^1/2^/W [5]. This device peak responsivity is 1.3 × 10^11^ cm Hz^1/2^/W at 5 µm and 0 V with − 10 V gate bias voltage which is comparable with the nBn device. But the weak point is device RA uniformity which influences device performance.

In conclusion, forcing the active region to be *p*-type through doping in the *π* region can effectively improve the performance for LWIR InAs/GaSb superlattice P^+^–*π*–M–N^+^ detector [6]. However, it is not the higher the doping concentration, the more the improvement in the device performance. Particularly, the electrical and optical performance of the device could be reduced by excessive doping concentration. Optical characterization at 77 K indicates that we obtain a QE’s maximum of 62.4% at 4.26 µm and a maximum of 2.26 A/W at 5 µm with Be doped with 780 °C. Electrical characterization suggests that a RA’s maximum of 1259.4 Ω cm^2^ with Be doped with 780 °C is obtained. The specific detectivity reaches its maximum of 5.6 × 10^10^ cm Hz^1/2^/W at 5 µm with Be doped with 780 °C. Moreover, the saturation bias of the device can be dramatically reduced through a simple and effective anodic vulcanization pretreatment. Vulcanization pretreatment exhibits its potential to reduce the gate bias voltage. Electrical characterization illustrates that the saturation bias is only − 10 V, while it is 40 V in other researches without vulcanization pretreatment in a similar structure with the same thickness of the SiO_2_ layer. Furthermore, the device performance can be significantly improved by applying a proper negative bias on the gate electrode. A maximum of 1.3 × 10^11^ cm Hz^1/2^/W is reached at 5 µm and 0 V with − 10 V gate bias voltage with Be doped with 780 °C at 77 K. Limited by experimental equipment and experimental conditions, we choose SiO_2_ to be dielectric layer, but in the follow-up, it is considered to use Hi–K medium for further experiments. Theoretically, gate bias voltage can be further reduced.

## Data Availability

The authors declare that the materials and data are promptly available to readers without undue qualifications for material transfer agreements. All data generated or analyzed during this study are included in this article.

## Abbreviations

LWIR: Long-wavelength infrared detectors
QE: Quantum efficiency
T2SLs: Type-II strained-layer superlattices
VLWIR: Very long-wavelength infrared detectors
MWIR: Middle-wavelength infrared detectors
HRXRD: High-resolution X-ray diffraction
AFM: Atomic force microscopy
ICP: Inductively coupled plasma
GD: Gated diode
UGD: Ungated diode
D*: Specific detectivity
RA: Product of resistance value and die area
Ri: Responsivity of infrared detector
G–R: Generation–recombination
